# The long way ahead to achieve an effective patient safety culture: challenges perceived by nurses

**DOI:** 10.1186/s12913-018-3467-1

**Published:** 2018-08-22

**Authors:** Jamileh Farokhzadian, Nahid Dehghan Nayeri, Fariba Borhani

**Affiliations:** 1 Nursing Research Center, Kerman University of Medical Sciences, Kerman, Iran; 2 School of Nursing and Midwifery, Tehran University of Medical Sciences, Nosrat St., Towhid Sq, Tehran, 1419733171 Iran; 3 Department of Nursing Ethics, Medical Ethics and Law Research Center, Shahid Beheshti University of Medical Sciences, Niyayesh Complex, Niyayesh Cross-Section, Vali-e-Asr St, PO Box: 1985717443, Tehran, Iran

**Keywords:** Quality improvement, Clinical risk management, Patient safety, Organizational culture, Qualitative content analysis

## Abstract

**Background:**

The **s**afety culture has recently attracted the attention of healthcare organizations. Considering the importance of the roles of nurses with regard to patient safety, their knowledge and experiences of the challenges that influence patient safety culture can facilitate the development and implementation of better strategies. The aim of this study was to explore the nurses’ experiences of the challenges influencing the implementation and integration of safety culture in healthcare.

**Methods:**

A qualitative study with deep and semi-structured individual interviews was carried out using a purposive sampling method to select 23 nurses from four hospitals affiliated with a large medical university in Southeast Iran. Data were analysed using the conventional content analysis of Lundman and Graneheim.

**Results:**

Data analysis reflected the main theme of the study, “A long way ahead of safety culture”. This theme includes four categories: 1) inadequate organizational infrastructure, 2) insufficient leadership effectiveness, 3) inadequate efforts to keep pace with national and international standards, and 4) overshadowed values of team participation.

**Conclusion:**

While practical strategies for creating a safety culture may seem simple, their implementation is not necessarily easy. There are several challenges ahead for cultivating an effective and positive safety culture in healthcare organizations. To keep pace with international standards, healthcare managers must employ modern methods of management in order to overcome the challenges faced by the institutionalization of safety culture and to make a difference in the healthcare system.

## Background

While patient safety is a new and emerging phenomenon, historical evidence indicates that concerns for patient safety have existed for a long time before modern healthcare. More than 150 years ago, Florence Nightingale stated that “the very first requirement in a hospital is to do no harm to patients” [1]. In 2001, the Institute of Medicine (IOM) issued this concern in the form of “Crossing the Quality Chasm: A New Health System for the 21st Century”. IOM emphasized the safety of healthcare and that patients should be free of danger or risk caused by the healthcare system [2].

Nowadays, modern advances and the complexity of healthcare have led to serious deficiencies in the quality of care and patient safety. The high prevalence of clinical risks and safety incidents have increased concerns and challenges for healthcare systems [3, 4]. Although estimates of the size of the problem are imprecise, it is likely that millions of people suffer from disabling injuries or death in consequence of clinical risk and safety incidents [5]. It is estimated that, in the hospitals of developed countries, one in ten patients is harmed while receiving unsafe care. Moreover, the risk of damage is much higher in developing countries than developed countries. For example, the risk of healthcare-associated infection in some developing countries is twenty times higher than that in developed countries [6].

However, the majority of injuries and safety-related deaths are preventable by designing and planning safety processes and techniques [5]. Therefore, to handle these challenges and to achieve quality and safety improvement, healthcare organizations are faced with increasing pressure to cultivate an effective safety culture [7]. Patient safety culture is defined as a dimension of organizational culture [8]. Specifically, it is the product of individual and group values, beliefs, attitudes, perceptions, norms, procedures, competencies, and patterns of behaviour that determine the commitment of an health organization to patient safety management [7, 8].

Safety experts have suggested the essential components for safety culture such as teamwork, leadership support, communication [7], and a just culture as well as a reporting and a learning culture [9]. Organizations with a positive safety culture have communications based on mutual trust, shared perceptions of the importance of safety, and confidence in the efficacy of preventive measures and support for the workforce [1]. Safety culture emphasizes preventive or predictive measures of safety more than retrospective ones [10]. In addition, the safety culture emphasizes the system approach or “why” unsafe acts occurred rather than the person approach or “who” made the unsafe acts [1]. By developing a system approach, hospitals should foster a continuous learning environment by reporting and discussing clinical risks and safety incidents without fear of punishment [7].

To promote a safety culture and to ensure safer healthcare systems, the IOM has emphasized the development of clear patient safety programs; use of non-punitive systems for reporting and analysing safety incidents; incorporation of well-established safety principles such as standardized and simplified equipment, supplies, and work processes; and establishment of interdisciplinary team training safety programs. However, the IOM has announced that the biggest challenge for moving toward safety culture is changing the culture from one which lays the blame on individuals to a system approach [11].

Institutionalizing a safety culture is the shared responsibility of all healthcare providers in the healthcare system [12]. Nevertheless, nurses have the central role in improving patient safety culture. When facing the challenges of healthcare systems, nurses are well positioned to protect the safety of patients or harm them with unsafe practices [13]. Therefore, both patients and nurses will be protected, a safe milieu for nurses will be created, and distress caused by safety incidents will be decreased by improving safety. Finally, these actions will reduce attrition and alleviate chronic nursing shortages [14].

However, creating a positive safety culture is challenging for healthcare systems, and some studies suggest that healthcare providers, especially nurses, give more problematic responses to patient safety culture [15, 16]. An Iranian study indicated that hospitals did not meet a proper level of patient safety and a punitive culture dominated the workplace [17]. A systematic review on six qualitative studies has demonstrated that hospitals underestimate organizational resources, and support is required for making patient safety initiatives and changing the culture [18]. In another systematic review, researchers attempted to address the interventions used to promote safety culture in healthcare, but they did not assess the challenges of safety culture [8].

World Health Organization (WHO) noted that, due to the multidimensional nature of safety culture, a better understanding of the factors influencing the patient safety culture and addressing the interventions to improve patient safety are research priorities in developing countries and countries with transition economies [5]. Additionally, Vlayen et al. mentioned that safety culture varies over time and this variation is linked with organizational context such as hospital statute and size and human resource characteristics such as educational background [19]. In this respect, the experiences of nurses as the most important human resources can help develop appropriate theories and provide specific guidance for healthcare professionals in facilitating safe practice in both developed and developing countries [13].

In-depth qualitative studies are required for a better understanding of factors impacting the development of patient safety initiatives and challenges facing the institutionalization of those factors [20]. Although some effort has been made to improve safety culture in Iranian hospitals, there is no comprehensive, qualitative study that explores the challenges of implementing and integrating a positive safety culture in the Iranian healthcare from the nurses’ perspective. The aim of this study was to explore and describe the nurses’ experiences of the challenges facing the implementing a positive safety culture within the culture and context of Iranian hospitals.

## Methods

### Study design

A qualitative and conventional content analysis with a descriptive-explorative approach was used for data collection and analysis. The qualitative content analysis method is the process of understanding, interpreting, and conceptualizing the underlying meanings of qualitative data. Although this method can be implemented with various degrees of interpretation including manifest messages versus latent messages, both messages require interpretations which may vary in depth and level of abstraction [21].

### Settings

The settings for this study were four teaching and referral hospitals affiliated with Kerman University of Medical Sciences in southeast Iran. With the population of about 800,000, Kerman is the largest city in this part of the country. These hospitals have more than 1000 beds in emergency, neonate, pediatric, surgery, and internal medicine, dialysis wards; and CCU, ICU, NICU, etc. to provide specialised care to patients with, among others, cardiac, endocrine, pulmonary, gastrointestinal, neurological, and psychological disorders. They receive patients from southeast of Iran, within the radius of 500 km 24 h per day, 7 days per week. Different high-tech medical and surgical interventions are conducted in these hospitals by nursing and medical staff who collaborate with university-based medical scientists in educating healthcare students.

The implementation of healthcare policies is centralized in Iran, and Ministry of Health governs all the hospitals [22, 23]. The political agenda is currently paying more attention to the reduction of patient harm, ensuring quality, and improvement of patient safety culture. To undertake this mission, all the hospitals are actively implementing “Clinical Governance” principles and “Hospital Accreditation Standards” [24]. In addition, the distribution of healthcare providers is similar in all the hospitals all over the country. The majority of nurses have Bachelor’s degrees and the recruitment of nurses follows the same pattern in all the hospitals [22].

### Participants and sampling

Purposeful sampling was used to select participants who were informed and experienced. In each hospital, a number of eligible participants were selected by the first researcher and assistance from the nursing managers. Nurses were recruited by written invitation informing them of the aims and methods of the study and asking them to indicate their willingness to participate and organize an interview session. The first interviews were done with a key informed participant, who had enriched experiences regarding various roles of a clinical nurse. The sampling process continued based on the principles of maximum variation to capture the rich and diverse perspectives and experience of nurses Therefore, 23 nurses with different backgrounds in sex, age, years of work experience, degrees in nursing, position, and type of ward were enrolled until data saturation. The nurses were 15 women and 8 men aging 26–50 years with work experiences ranging from 1.5 to 26 years. Six nurses had a master’s degree and the others had a bachelor’s degree in nursing. Seven participants came from hospital 1, six from hospital 2, five from hospital 3 and five from hospital 4. The sample included a matron, three clinical supervisors, an educational supervisor, four head nurses, a quality improvement officer, and thirteen clinical nurses. Having at least bachelor’s degree in nursing, the work experience for at least one year, and willingness to participate in this study were the main inclusion criteria employed for the selection of the participants.

### Data collection

Semi-structured, individual, face-to-face, in-depth interviews were carried out by one of the researchers who had a background in clinical nursing and patient safety work. Interviews were scheduled after explaining research objective to nurses. All interviews were initiated with an open question asking the participants to describe their patient safety work. The interviews were directed towards the purpose of the study by topic guide questions, e.g. “Would you please describe your experience with integrating safety culture in organizational culture?”, “To your experience, what requirements were necessary for safety culture in your hospital?”, “What activities have been carried out in improving the safety culture in your hospital?”, and “What barriers and challenges have you experienced for implementing these activities?”. Then, probing questions were asked to obtain more elaborative answers and nurses’ experiences, e.g. “Would you explain the situation clearly?” We considered patient safety in simple terms such as the prevention of errors and adverse effects to patients associated with healthcare.

The interviews lasted for 50–75 min and took place in a quiet room near the ward or in another location based on the participants’ preference. All interviews were audio-recorded with the participants’ permission and transcribed verbatim using Microsoft Office Word. The information was saturated after twenty-one interviews and two additional interviews were conducted to ensure data saturation.

### Data analysis

Data analysis was conducted according to the method proposed by Graneheim and Lundman [25]. In this approach, iterations consisted of the following: In the first step, every interview was transcribed verbatim by the first researcher, the researchers read the transcriptions several times to obtain an overall understanding of the content, and performed an initial coding individually. Second, the text was divided into meaning units that were then condensed. Each meaning unit comprised words and sentences containing aspects related to one another. Third, the condensed meaning units were abstracted and labelled using open codes. In the fourth step, the codes were classified into subcategories and categories based on similarities and differences. A category consists of similar codes in the manifest level. Finally, the underlying meaning and content of the data were extracted, and themes were formulated as the expression of the latent meaning of a text. All researchers discussed the content of the categories using triangulating analysis. When the authors disagreed, discussions and clarifications continued until achieving a consensus.

### Trustworthiness

The trustworthiness of the data was determined using Lincoln and Guba’ criteria, including credibility, confirmability, dependability, and transferability [25]. To enhance credibility, the researcher established a friendly relationship with the participants and had prolonged engagements with the research settings and data analysis (in the 7-month period from April to October 2016). Moreover, the data were initially coded and categorized independently by the researchers and the developed themes were compared. Where there was disagreement, discussions were held to reach consensus among all three co-authors. For member checking, a summary of the interviews and results was presented in prescheduled meetings with ten participants. They confirmed the contents as accurate and that the researchers were representing their real experiences and perspective; only three of them suggested minor changes to the situations and wording of codes. However the areas of disagreement were discussed, and feedback loops were used to ensure rigour. New codes were added and some codes were eliminated. The confirmability and dependability of data were assessed through member checks by colleagues and participants. In addition, external checks were carried out such that the first author met 5 nurses, 3 senior managers, 2 physicians who had not participated in the study and were informed of patient safety, and members of the patient safety committee in hospitals who confirmed the accuracy of our findings. In addition, research and decision-making processes were accurately recorded and reported so that the follow-up by others and data verification would be possible.

## Results

### The long way ahead of safety culture

Table 1 presents one main theme, four categories, and 16 subcategories that were perceived by nurses as challenges of implementing and integrating safety culture in healthcare. According to the newly emerging safety culture in Iranian hospitals, there are many challenges for establishing a safety culture. Due to these challenges, there is a long way to integrate an effective safety culture in the organizational culture. They also have a negative impact on nurses in safety programs. Results are explained in the following sections using the direct quotations of the participants.

**Table 1:** The main theme, categories, and subcategories

Main Theme	Categories	Subcategories
The long way ahead of safety culture	1. Incompetent organizational infrastructure	- Shortage of resources - Weakness of the staff’s professional competence and empowerment - Unfavourable work condition and unsafe environment
	2. Insufficient leadership effectiveness	- Lack of commitment - Non-supportive management - Non-participatory decision-making - Low efficiency of safety management rounds and clinical audit
	3. Inadequate efforts to keep pace with national and international standards	- Failure to establish quality improvement and clinical risk management systems - Culture of resistance to change towards the system approach - Culture of blame and punishment - Weakness in feedback to reporting errors - Weakness in the culture of organizational education and learning -Weakness in preventive (protective) culture
	4. Overshadowed values of team participation	- Failure to redefine and clarify roles - Gap in team coordination - Difficult dynamics of team interactions

### Incompetent organizational infrastructure

According to participants’ opinion, the risk of harm to patients, staff, and organizations has been increased by some factors, including “shortage of resources”, “weakness of the staff’s professional competence and empowerment”, and “unfavourable work condition and unsafe environment”. These factors have also hindered the institutionalization of an effective safety culture.

#### Shortage of resources

Safety management programs and strategies of safety improvement have been affected by the shortage of financial and human resources, medical supplies, medications, medical devices, and technology. Moreover, a lack of resources has led to failure in the innovation of safety programs. A quality improvement officer with 21 years of experience stated:

“Although the first action for an organization's safety culture is to construct the organization infrastructure, the implementation of safety improvement strategies has been blocked by insufficient funds, inadequate human resource, and unsafe physical environment.”

#### Weakness of the staff’s professional competence and empowerment

According to the experiences of nurses, the institutionalization of safety culture has been challenged by some factors, including a lack of creativity, inadequate professional and moral competence, lack of ability in judgment and clinical decision-making, lack of knowledge and ability in care management, and lack of effort for improving professional competencies. An educational supervisor with a master’s degree and 19 years of work experience indicated:

“Although all our staff have to be safety managers, they are not capable because safety training courses have failed to properly improve their safety competence. On the other hand, transferring responsibility to employees without necessary qualifications and competence increases the clinical risk.”Some nurses believed that newly graduated nurses do not develop adequate capability and competence for patient safety because there is no coherent curriculum in the field of patient safety in nursing education, especially in clinical training in university. One of the newly hired nurses from the surgery ward with 1.5 years of work experience said:

“Learning was not enough in the university. When I started working in the hospital, I realized that the training and courses on patient safety were highly inadequate at university, and I came to know the safety culture in the workplace.”

From another perspective, participants with more work experience believed that even experienced nurses might endanger the patients’ safety due to various reasons such as false self-confidence, unwillingness toward change, and lack of up-to-date competencies. Therefore, all the staff involved in healthcare should acquire up-to-date competencies regarding their responsibilities in order to create an effective safety culture. Furthermore, they should receive effective, efficient, and specialized training proportionate to their position and responsibilities.

#### Unfavourable work atmosphere and unsafe environment

Nurses need to have proper conditions and environment at the workplace in order to provide safe care for patients. The strengthening of safety culture is difficult to achieve at the workplace because of factors such as lack of time, hard and boring work, high level of patient admission and discharge, poor communication with co-workers, environmental stress, emotional pressure after patients’ harm or death, and tension in the patients’ families. A headnurse from the emergency ward with 16 years of work experience stated:

“A crowded ward and a high number of patients threaten the safety of staff and patients. The staff are under pressure and also at risk by agitation and high levels of workload and tension. Therefore, their views on the safety culture would be negative in such stressful conditions.”

### Insufficient leadership effectiveness

This category includes four challenges: “lack of commitment”, “non-supportive management”, “non-participatory decision-making” and “low efficiency of safety management rounds and clinical audit”.

#### Lack of commitment

The safety culture is established when the leaders have committed themselves to creating, shaping, and transferring it to the workplace. The integration of safety culture to organizational culture will never be successful without thoughtful, transformational, and committed leaders. According to the experiences of nurses, the leaders do not have commitment or creativity to strengthen the safety culture due to a number of factors, e.g. ineffective policies, poor-quality supervision, poor leadership practices, absence of resource allocation, lack of appropriate measures for promotion and giving rewards to employees, and lack of integrated management in the field of safety. A clinical nurse from the dialysis ward with 23 years of work experience said:

“The managers’ supervision is based on their opinions and they do not have a specified instruction for monitoring. For example, some managers are patient-centred and they walk round the ward with the view of patient safety. On the other hand, some managers do not have a clear recognition or intuition of the basic principles, operational aspects, and requirements of safety culture. Consequently, we will be confused if our managers are not committed to the guidelines of safety.”

The nursing managers also approved these challenges and considered continuous and precise supervision as one of the major managerial roles of managers committed to the safety culture. A nursing manager with 26 years of work experience expressed:

“Commitment of the managers to safety culture and precise supervision is the fundamental principle of continuousness of safety, but unfortunately some of the managers lack the sufficient commitment and effective supervision.”

#### Non-supportive management

According to the experiences of nurses, the support of employees is an important step for effective leadership in safety culture. Managers should pay attention to psychological, mental, and emotional supports of their staff, and their supportive behaviour motivates the nurses to promote their capabilities and implement safety programs. In addition, nurses mentioned a number of challenges, including managers’ lack of attention to workplace pressures and the heavy duties of nurses; managers’ discrimination, aggression, and violence towards the staff; and the lack of support they had experienced in this field. A clinical nurse from the orthopedic ward with 5 years of work experience reported:

“In our ward, a patient had cardiac arrest immediately after receiving an injection. The nurse tried to defend herself, but she was not successful. Thereafter, she was not supported and was thus punished. In the meeting for root cause analysis of events, managers did not allow the nurse to give a clear and precise report about that event.”

However, some managers had an opposite view. They believed that, in the event of errors, they would attempt to support their staff and even introduce them to the nursing system and nursing association. A clinical supervisor with 20 years of work experience stated:

“In case of system error, managers provide their staff with emotional support. But in case of negligence in performing duties, the managers don’t support them because such cases are often very complicated and it is impossible to support the staff.”

#### Non- participatory decision making

Nurses believed that individuals who have to perform safety rules should participate in the development and review of the rules. Nurses’ point of view on effective leadership has been changed negatively because of their dissatisfaction with lack of participation in decision-making for safety matters. Consequently, those conditions were a barrier to their active participation and movement towards the establishment of a safety culture. A nurse from the emergency ward with 10 years of work experience and a master’s degree said:

“The physical space of the emergency department was changed, but the opinions of nurses and supervisors were not considered. Now, the beds are so close to each other, and if a patient suffers from cardiac arrest, there will be no feasibility for cardiac and pulmonary resuscitation and for taking the emergency trolley beside the patient’s bed.”

#### Low efficiency of safety management rounds and clinical audit

Participants believe that organized planning for targeted walk rounds as well as observing and talking directly with staff are the best ways for senior managers to become familiar with safety activities and discover the causes of large safety problems. This method is much more effective than the inspection of statistics and reports in the office. Although the majority of managers believed that they underlined the necessary importance of safety issues in walk rounds, the reaction of nurses shows that safety walk rounds did not have the required output. A clinical nurse from the surgery ward with 13 years of work experience noted:

“Some managers believe that the commitment of management to safety only includes the preparation of protective equipment, installation of mottos, safety signs, punishment, reward, and preparation of statistics. If managers listen closely to the problems and concerns for employees in regular and targeted walk rounds and track corrective actions, the staffs will be assured that managers have fulfilled their promises. But unfortunately this has not happened and walk rounds have not been operational and had no appropriate output yet.”

The nurses introduced clinical audit as an important way to evaluate and enhance the safety culture using standard checklists. Nevertheless, they believed that the process of audit has failed for a number of reasons, such as the lack of motivation of the audit team due to receiving no reward, lack of expert team, and putting no importance on the audit by staffs and managers. A clinical supervisor with 23 years of work experience in different wards stated:

“Sometimes, individuals who perform the audit are not good or experts in auditing. Moreover, when the results of the audit are used in the safety part, we see that no improvement has been made in the next period of the audit.”

### Inadequate efforts to keep pace with national and international standards

Quality improvement systems, e.g. accreditation, clinical governance, and clinical risk management, are strategies for keeping pace with national and international safety standard. In addition, these strategies provide the convergence and alignment of healthcare providers for reaching a safe care and establishing safety culture all over the world. The data showed that the efforts are not adequate for integration and adjustment with these strategies. A number of subcategories were: “failure to establish quality improvement and clinical risk management systems”, “culture of resistance to change towards system approach”, “culture of blame and punishment”, “weakness in feedback to reporting errors”, “weakness in the culture of organizational education and learning”, and “weakness in protective culture”, reflecting the limited and disconnected efforts of all levels of healthcare providers.

#### Failure to establish quality improvement and clinical risk management systems

The participants believed that quality improvement systems have taken good measures to implement safety management, such as infection control, but hospitals are far from standards. Based on the perspective of nurses, challenges, e.g. lack of knowledge, problems of management and organizational culture, limited human and financial resources, high workload, lack of time, and weakness in team work, are the causes of staff’s negative attitude towards the approaches of quality and safety improvement. A head nurse from the surgery ward with 19 years of work experience said:

“Most of the staff oppose to clinical governance and accreditation. They say that these issues have multiplied the workload of nurses by a hundred times, because managers did not create an appropriate substructure and did not provide an infrastructure for them. Rather, they just want the nurses to perform those tasks. Therefore, the communication of nurses with patients has been lowered, and this is the cause of a drop in the quality and safety of services.”

#### The culture of resistance to change towards a system approach

To strengthen the safety culture, the system approach is required. The system approach helps managers to comprehensively review safety events and prevent the accidents arising from system’s problems by reforming the system’s defects. Therefore, changes have to be applied to the organizational culture, structures, systems, procedures, environment, equipment, processes, and organizational behaviour. However, according to nurses’ experiences, there is resistance against culture change. Even managers are not aware of the social, human, and economic benefits of the system approach. A quality improvement officer with 21 years of experience stated:

“We need to change our vision from an individual to a system approach. Hence, when a hazardous accident happens, we should search for the defect in the system rather than the punishment of an individual. For example, when a high-risk medication was prescribed by mistake, we noticed that there was no instruction for the injection of the high-risk medication in the ward and the nurse was not guilty.”

According to some participants, although the managers pretended that they had a systemic view of errors, that is, in fact, not the case. A clinical nurse with 10 years of work experience and contractual employment in the medical ward said:

“In meetings, managers tell us to brainstorm and report the errors without fear, but in practice, they act in the opposite way because they do not yet have a systemic view. Once I reported a completely systemic error, but they immediately warned and threatened to fire me and cancel my employment contract.”

#### Culture of blame and punishment

In order to reduce safety incidents, special attention should be paid to the culture of errors and safety event reporting as a key component of safety culture. Furthermore, the culture of punishment and blame-seeking should be avoided. Nurses enumerated the following factors for the culture of blame; the fear of being blamed and punished, disregarding the nurses’ defence, reprimand and disciplinary measures. A clinical supervisor with 23 years of work experience noted:

“The statistics of safety incident reporting is low in our hospital because employees are worried about the consequences of reporting, such as reproaching, rebuking, and jeopardizing their jobs. The failure to report the incidents may underestimate statistics of incidents. However, it does not enhance the safety culture.”

#### Weakness in feedback to reporting errors

The system of receiving data and reporting error is obliged to perform a radical analysis of safety events, analysis of findings, diffusion of corrective actions, and provision of feedback for staff. Nurses believed that the lack of some actions, e.g. report delivery, feedback to staff, and follow up of their feedbacks, indicates the lack of importance of reporting and weakness of safety culture. A clinical nurse from NICU with 3 years of work experience and a master’s degree stated:

“We completed lots of error reporting forms and sent them to the office of quality improvement, but we did not receive any feedback or corrective action. Thus, we conclude that reporting has no benefit. In addition, actions without consequent monitoring, evaluation, and feedback would not create long-lasting changes in safety.”

Although some of the managers believed that their goal of error reporting is to provide the staff with appropriate feedback and learn from errors, some of the nurses believed that the managers’ viewpoint is one-sided.

#### Weakness in the culture of organizational education and learning

Teaching and learning individually, institutionally, or in group are stimulants for system approach and strengthening the safety culture. The culture of learning from past incidents will improve safety function and prevent the repetition of those incidents. According to the experiences of nurses, the staff has lost motivation for organizational learning because of their passive presence in training courses, lack of appropriate training, lack of time, and high workload. A clinical nurse form CCU with 11 years of work experience mentioned:

“There is no appropriate planning for training, and the intention of managers is to show that a class has been held because it is mandatory for them to arrange risk management and safety classes for receiving accreditation. The classes are not efficient, and the managers only fabricate documentation.”

Moreover, the participants with master’s degrees stated that nurses who have attempted to promote their educational degrees have never seen the outcome of their efforts appropriately. These nurses believed that some factors play a major role in reducing their motivation for organizational learning, e.g. ignoring their knowledge and capabilities as the major members whose activities influence the safety culture, as well as the nursing managers’ deliberate efforts to prevent the nurses’ individual promotion.

#### Weakness in preventive (protective) culture

Nurses believed that staff and managers should not expect an incident to happen and then take corrective actions. Sometimes this immoral approach is at the cost of harming the patients and employees. Therefore, managers need to support the preventive approach to strengthen the safety culture. They should also be a role model for the staff. “Failure modes and effects analysis”)FMEA(is a preventive (protective) approach in safety, but no improvement has happened in this field in the hospitals. Moreover, the preventive culture has not entered most processes yet. A clinical supervisor with 19 years of experience said:

“When a fire happened due to system malfunction in our emergency room at midnight, it caused an accident for patients and staff. Thus, if preventive measures had been taken, we would not have spent subsequent costs. We should always have a strong preventive insight, but we have considerable weakness in this area.”

### Overshadowed values of team participation

Safety culture requires teamwork at all levels of organization and at every level of education and skill, and it will not be possible without team participation and intellect. The values of team participation in the establishment of a safety culture are overshadowed by a number of challenges, including “failure to redefine and clarify roles”, “gap in team coordination”, and “difficult dynamics of team interactions.”

#### Failure to redefine and clarify roles

According to nurses, clarity in the roles of healthcare team is very important to the promotion of patient safety. However, factors such as lack of clarity in duties and roles in the team, resistance to the implementation of roles and responsibilities, lack of education about the roles, and lack of understanding of one’s place in teamwork inhibit the effective roles in the team, thereby threatening safety. A head nurse from the surgery ward with 19 years of work experience stated:

“Some members of the healthcare team do not agree with their responsibilities in risk and safety management (e.g. identifying risks and reporting incidents). According to them, these responsibilities are not parts of their duties. Therefore, risk and safety management should be clarified in the job description of each member of the healthcare team.”

The participants with a master’s degree believed that despite the improvement of their academic level, their duties and roles have not been changed, so that they still have to continue to work in their former job position. Therefore, continuing education at higher levels is not an advantage or a guarantee to obtain an appropriate job position. It might even lead to more duties and responsibilities for nurses with a Master’s degrees.

#### Gap in team coordination

From the viewpoint of nurses, coordination is an important aspect of team participation. They believe that physicians are the most important group that impose risk on the patients. On the other hand, physicians resist when they are invited to participate in safety team programs, such as root cause analysis, or they do not have coordination with therapy and management teams in many care and treatment programs. A nursing manager with 26 years of work experience said:

“The physicians’ coordination and participation in safety programs is very low. The committee of root cause analysis of events invited a resident for the radical analysis of an error, and the resident opposed the committee and refused to attend the meeting.”

#### Difficult dynamics of team interactions

Participants acknowledged that positive interactions and professional relationships between them and their colleagues, nurse leaders, and physicians working as a team would promote the safety culture. They emphasized that appropriate relationships create a favorable emotional, psychological, and social atmosphere and reduce their stress in the work environment. Nevertheless, factors such as unprofessional and inappropriate communications and lack of knowledge of communication principles have created difficult dynamics of team interactions. In such situations, nurses would have unfriendly feelings that negatively affect their performance and decrease patient safety. A clinical nurse from the emergency ward with 7 years of work experience indicated:

“Some of my colleagues have forgotten the purpose of reporting safety events. They complain about the reports given by other colleagues. These behaviors spoil professional communication and show no honesty between nurses.”

According to nurses, nurse leaders in a multidisciplinary team have a vital position to improve the dynamics of team interactions. Therefore, the nurses expected from nurse leaders to facilitate professional relationships, encourage multidisciplinary collaboration, and resolve communication difficulties between nurses and other healthcare providers. However, some leaders do not have appropriate communication with nurses and do not understand their concerns about patient safety. Such ineffective communication discourages nurses from taking patient safety measures such as reporting their errors.

In terms of emphasizing the influence of all healthcare team members, the nurse–physician communication was an important factor which influenced patient safety. The nurses believed ineffective communication between physicians and nurses could act as an obstacle to improved patient safety culture due to perceived hierarchical differences. In addition, physicians’ individualism and their attitudes have prevented them from accepting nurses’ opinions and ideas or using their knowledge and experience in decision-making. Such conditions have had negative impacts on the performance of nurses and caused stress, lack of motivation, and lack of sense of mental security among the nurses. A clinical nurse from ICU with 13 years of work experience and a Master’s degree stated:

“The relationship between physicians and nurses is not desirable. Some physicians act individually and do not examine nursing reports. They seldom ask nurses about patients conditions or whether patients have improved with the previous day’s prescription. Furthermore, physicians hardly accept our suggestions.”

## Discussion

The present study attempted to attain an insight into the challenges of establishing an effective safety culture using the experiences of nurses. These challenges have affected the performance, conception, and attitude of nurses to involve in safety culture and have slowed the movement towards an effective safety culture. The main theme of this study indicates that the healthcare system has a long way ahead to achieve an effective and positive safety culture.

One of the categories of this study was incompetent organizational infrastructure along with a number of subcategories, including the shortage of resources, unfavourable work condition and unsafe environment, and weakness of the staff’s professional competence and empowerment. These results were in agreement with previous studies [26–28] which reported that lack of human resources, financial, time, equipment, and information technology was the main obstacle to implementing quality improvement and patient safety programs. In a qualitative study in the UK, human resources were considered as having a key role in the process of changing the culture. On the other hand, it was reported that the implementation of safety programs will be difficult due to limitations in financial resources for human resource management (i.e. training, recruitment, and empowerment) and the lack of qualified, skilled, and trained personnel [29]. According to the results, nurses in general and newly graduated nurses in particular believed that nurses do not develop an adequate capability and competence for patient safety in university. In agreement with these results, Vaismoradi et al. [30] confirmed that new graduates experience stress as they become healthcare professionals, and that nursing education does not seem to prepare nurses for complex and challenging work environments.

This study is consistent with other studies showing that the work condition and environment, e.g. mental and emotional setting, profession attrition [31], working in shifts and fatigue [32], lack of control over complex and unsafe working conditions [33], high workload, crowded and irregular environment, and inadequate space [27] affect patient safety and quality improvement. Mahmood et al. [34] emphasized that inappropriate and unsafe work environment cause physical and mental pressure and stress for nurses, increasing the risk of accidents. They also indicated that factors, including comprehensive understanding of environmental factors, considering interventions and appropriate strategies for managing the challenges of physical environment, and mental and emotional settings, are prerequisites for managing the safety culture.

In the present study, insufficient leadership effectiveness along with some challenges, such as lack of commitment, non-supportive management, non-participatory decision-making, and low efficiency of safety management rounds and clinical audit, were significant challenges for the institutionalization of the safety culture. These findings are in agreement with the results of other studies showing that the programs of quality improvement have faced some challenges, including a lack of leadership commitment and inefficient management [35], flaws in involving employees in safety programs, and lack of autonomy and a supportive setting [36]. In another study, researchers acknowledged that when nurse leaders welcomed nurses’ participation in decision-making, asked for their perspectives, devolved the responsibility and authority of managerial duties, and administered appropriate supervision, nurses responded to the leaders’ trust by becoming more committed to patient safety [37].

Low efficiency of safety management rounds and clinical audit was mentioned as one of the challenges in the present study. In a study in Iran, numerous obstacles were reported for the effectiveness of clinical audit and safety walk rounds. These obstacles were insufficient allocations of resources, inadequate standards for the audit, lack of trained personnel, shortage of personnel for follow-up safety measures, incomplete documentation, and low collaboration of clinicians in the audit process [38]. Contrary to this study, however, safety management rounds were introduced as an effective tool in promoting risk management activities and safety culture in a mixed method study. These visits increased the commitment and accountability of senior management to the safety of patients, employees, and the society [39]. In addition, clinical audit and feedbacks of the audit were mentioned as an important strategy for the continuation of quality and safety improvement activities in a systematic review [40]. This difference is probably due to the inadequate organizational infrastructure of the hospitals mentioned in this study.

Failure to establish quality improvement and clinical risk management systems was another challenge for the effective safety culture. In this regard, a study in Iran reported similar challenges and obstacles, including limited financial resources and equipment, human resource constraints (e.g. lack of skilled workers and low motivation), management problems, weakness in training programs, failure in communications, cultural issues (e.g. feeling no need to change and lack of team participation), and laws and policies (e.g. the absence of harmonized rules and poor assessment of the Ministry and universities) [35]. These suggest that progress in patient safety and quality improvement systems is still far from ideal in Iran. One of the important roots of this situation might be the failure to address the patient safety system in scientific, academic, and research gatherings, as well as the failure to provide proper infrastructure to implement them.

The culture of resistance to change towards a system approach was mentioned as one of the safety culture challenges. In a study, the political context of healthcare, lack of required skills among managers and the pressure to deliver quick and measurable changes were reported as barriers to changing the culture of quality and safety programs in [41]. Another study reported that, with respect to the complexity of organizational factors such as culture, structures, and processes, change in the organizational fundament would not be easy but would be possible. Therefore, it is necessary to identify potential strategies for change [26]. This study highlights that change in the organization, creation, and promotion of safety culture and quality improvement is impossible without the support and involvement of different levels of management and leadership or without providing organizational infrastructure.

Regarding challenge of the culture of blame and punishment, in agreement with present study, a study in Iran reported that non-punitive response to errors is weak in hospitals. The Iranian healthcare context must avoid the culture of “name, shame, and blame” and implement the system approach [15]. In another study, factors related to the organization, e.g. the absence or lack of a safety culture, a culture of blame and reprimand, lack of support of the staff in the event of an error, an inappropriate function and reaction of managers, have been considered as barriers to error reporting [42]. The culture of blame is a barrier to reporting the errors and learning from mistakes. It also restrains the nurses from patient protection [43].

Weakness in feedback to reporting errors and weakness in the culture of organizational education and learning were the other challenges presented in this study. In agreement with this results a qualitative study in Australia which introduced the lack of receiving feedback from an error report, culture of blame, lack of value in the process, and failure to receive legal privileges as barriers to reporting the errors by nurses and doctors [44]. In organizational learning, the emphasis is on learning from errors and mistakes, providing an opportunity to learn later. One method to improve patient safety is to lead the organization towards organizational learning by enforcing the appropriate setting for learning from errors and their proper management [45]. Perhaps the reasons of weakness in organizational learning could be lack of learning conditions. For example, in a qualitative study by Heidari et al. [46], the lack of a sense of attachment, confidence, and satisfaction with professional position were identified as the main concerns of nurses, especially those with a master’s degree, in the process of organizational learning. Also, the nurses highlighted issues such as lack of confidence at the obtained competencies, deficiency in work autonomy, lack of decision-making power, absence of autonomy to use their knowledge, and predominance of physicians’ opinions which had deterrent effects on the efforts of nurses to achieve personal and organizational promotion. The interesting point about the experiences of nurses was that, in the current system, higher knowledge and skills were not considered as benefits for nurses, even creating more duties and responsibilities for them.

The participants believed that the weakness in preventive culture was another challenge for the safety culture. This study was consistent with a study which concluded that the errors receive attention by a more passive and retrospective (reactive) approach and people are considered as the cause of errors in Iran. In addition, the researchers in that study reduced the incidence of errors in the processes of emergency department by using the prospective method of “failure modes and effects analysis”)FMEA). They introduced the FMEA as an efficient and effective technique for the advancement of system thinking and access to safe processes of patient care [47].

Based on the results, challenges such as failure to redefine and clarify roles, gap in team coordination, and difficult dynamics of team interactions have overshadowed the values of team participation in the establishment of a safety culture. Castro-Sánchez et al. [30] highlighted that the consequences of lacking awareness about professional responsibilities (own and others’) could threaten patient safety. Various studies have reported similar challenges and obstacles to team participation in patient safety programs, risk management, and quality improvement. Those challenges were inconsistency in team communication, negative attitude to teamwork, unwillingness to perform duties in the team [48], lack of job descriptions [13], gap in team engagement and coordination [49], inadequacy in team communication and interaction [50], and lack of a culture of teamwork [42].

The participants in our study commented that an aspect of difficult dynamics of team interactions was the unfavorable relationship between nurses and physicians, leading to dissatisfaction among nurses and reduced patient safety. In consistent with the results other studies concluded that ineffective relationships affected nurses’ performance negatively and reduced care quality. Nurses reported that unfavorable relationships among some physicians and nurses disappointed the nursing staff, making them lose their motivation. In addition, lack of attention to nurses’ knowledge and capabilities by some physicians caused stress and unpleasant feelings regarding the work atmosphere, acting as a barrier to the proper delivery of nursing care [51]. Disharmony in the interaction between physicians and nurses will cause conflicts among the members of healthcare team, resulting in error and disruptions in patient safety. Therefore, improving the interactions between nurses and physicians should be considered as one of the effective strategies to improve patient safety.

### Limitations

This study explored merely a part of the challenges of institutionalization of an effective and positive safety culture based on the experiences of one professional group (nurses) in cultural and social fields in the hospitals of Iran. Therefore, the transferability of findings should be considered with caution and critiqued and compared with those of similar studies conducted in other contexts. In addition, others challenges may be recognized in different cultural and organizational contexts, with other groups of healthcare system, e.g. physicians, and even from a multidisciplinary perspective. Thus, more qualitative and quantitative studies must be performed to adopt the strategies and processes of the management of safety challenges proportionate to diverse cultural and organizational contexts. The results of such comprehensive research could pave the way for creating and institutionalizing a growing safety culture, leading to high quality and safe care in the unique cultural context.

## Conclusion

Despite the numerous efforts of healthcare system, there is still a long way towards the institutionalization of an effective and positive safety culture. Several challenges have slowed the realization of safety culture in the organizational culture. The patient safety culture is an international phenomenon and the challenges detected in this study are somewhat similar to other studies. However, adopting strategies and processes for the integration of safety culture in the cultural and organizational field should be consistent with underlying conditions which are unique in each hospital. More efforts are required in order to have an effective and positive safety culture in all parts of the organization, including management and healthcare staff. Quality improvement systems, such as accreditation, are useful tools for improving the safety culture. Healthcare managers should allocate the necessary resources to keep pace with these systems and to draw the participation of all members of the healthcare team. Moreover, they should employ social and cultural beliefs and capabilities to achieve an effective safety culture. It is also essential that they use modern management styles, such as change management and participative management, in order to make a difference in healthcare, overcome challenges, and establish the safety culture. It is suggested that more quantitative and qualitative studies be conducted to evaluate the strategies of overcoming barriers to the establishment of the safety culture.

## Abbreviations

CCU: Coronary care unit
FMEA: Failure modes and effects analysis
ICU: Intensive care unit
IOM: Institute of Medicine
